# Molecular surveillance revealed no SARS-CoV-2 spillovers to raccoons (*Procyon lotor*) in four German federal states

**DOI:** 10.1007/s10344-022-01605-7

**Published:** 2022-08-05

**Authors:** Ibrahim T. Hagag, Torsten Langner, Martin H. Groschup, Markus Keller

**Affiliations:** 1 Institute of Novel and Emerging Infectious Diseases, Friedrich-Loeffler-Institut, Federal Research Institute for Animal Health, Suedufer 10, 17493 Greifswald, Insel Riems Germany; 2 Faculty of Veterinary Medicine, University Leipzig, An den Tierkliniken 1, 04103 Leipzig, Germany

**Keywords:** SARS-CoV-2, Raccoons, RT-qPCR, Natural infection

## Abstract

Raccoons (*Procyon lotor*), which are closely related to the family Mustelidae, might be susceptible to natural infection by SARS-CoV-2. This assumption is based on experimental evidence that confirmed the vulnerability of farmed fur-carnivore species, including *Procyon lotor* to SARS-CoV-2. To date, there are no reports of natural SARS-CoV-2 infections of raccoons in Germany. Here, we use RT-PCR to analyze 820 samples from raccoons hunted in Germany with a focus on 4 German federal states (Saxony-Anhalt, Thuringia, Hesse, North Rhine-Westphalia). Lung tissues were homogenized and processed for RNA extraction and RT-qPCR for detecting SARS-CoV-2 was performed. No viral RNA was detected in any samples (0/820). Next, we compared raccoons and human ACE-2 residues that are known to serve for binding with SARS-CoV-2 receptor binding domain (RBD). Interestingly, we found only 60% identity on amino acid level, which may have contributed to the absence of SARS-CoV-2 infections in raccoons. In conclusion, the chance of raccoons being intermediate reservoir hosts for SARS-CoV-2 seems to be very low.

## Introduction

The raccoon (*Procyon lotor*) is a nocturnal omnivorous mammalian species that was introduced in Germany originally from North America in 1835. However, due to animals escape or release in the wild, their number is exponentially increasing, which make Germany one of the focal points of the European raccoon population (Pagel and Spieß 2011; Leicht 2009; Niethammer 1963; Stope 2019). This population surge could be attributed to their ability to adapt to different habitants, utilize a wide range of animal and plant food, highly reproductive potential, and the significant decrease in natural predators in Europe. Consequently, wild raccoons successfully colonized suburban and urban habitats, and are living in a proximity to humans and their pets, eat garbage and come even into contact with sewage. In North America, wild raccoons are either intermediate or final hosts for multiple zoonotic pathogens. Nonetheless, little is reported about the pathogen spectrum in the German and European invasive raccoon species (Bharti et al. 2003; Beltrán-Beck et al. 2012). The raccoon population in Germany is genetically heterogenous (Fischer et al. 2015; Biedrzycka et al. 2014), and therefore populations in close proximity may even show a different pathogenic pattern (Stope 2019). This is indeed a potential risk and therefore surveillance of pathogens, especially zoonotic pathogens, in wild raccoons in Germany and Central Europe is inevitable (Stope 2019).

In 2019, severe acute respiratory syndrome coronavirus-2 (SARS-CoV-2) emerged and caused a global pandemic (Zhu et al. 2020; Chan et al. 2020). Soon after its first appearance, a link to a wet market in Wuhan has been suggested as the origin of the transmission of the virus to humans, suggesting that a wild animal may have been the intermediate host of this original bat virus (Aguirre et al. 2020). Among animals, carnivores especially felids and mustelids are ultimately vulnerable to SARS-CoV-2 (Shi et al. 2020). For instance, ferrets are used as experimental model for SARS-CoV-2 and natural infections have been reported in farmed mink which even retransmitted the virus back to humans in some case (Munnink et al. 2021; Cahan 2020). Procyonidae are a closely related family to mustelids; however, little is known about their susceptibility to SARS-CoV-2. Coronavirus infections were reported in raccoons as a juvenile raccoon in the USA succumbed to infection and positive antibodies were detected against in raccoons in Japan (Martin and Zeidner 1992; Aoki et al. 2017). Raccoon dogs may have played also a role in the transmission chain of SARS-CoV-1 in 2003 (Guan et al. 2003). For this reason, and because of the zoonotic nature of coronaviruses, surveillance of SARS-CoV-2 infections in musteloid species was evident. Given the high potential of SARS-CoV-2 viruses for mutated variants, the potential of reverse zoonotic infections to wildlife species is certainly also an issue. Moreover, co-infections of species-specific coronaviruses and SARS-CoV-2 might lead to novel chimeric viruses (Focosi and Maggi 2022; Banerjee et al. 2020; Boniotti et al. 2016). Until now, experimental studies showed that raccoons are not susceptible to SARS-CoV-2, since challenged animals did not develop clinical symptoms nor shed virus genomes or infectivity (Francisco et al. 2021; Bosco-Lauth et al. 2021). Nonetheless, a variety of factors that are difficult to simulate in experimental studies such as coinfections, immunodeficiencies, gestation, senescence, parasitic burdens, and environmental conditions may also contribute to their susceptibility. Therefore, searching for natural SARS-CoV-2 infections in raccoons and wildlife using a screening approach will provide supportive answers.

## Materials and method

**Fig. 1:**
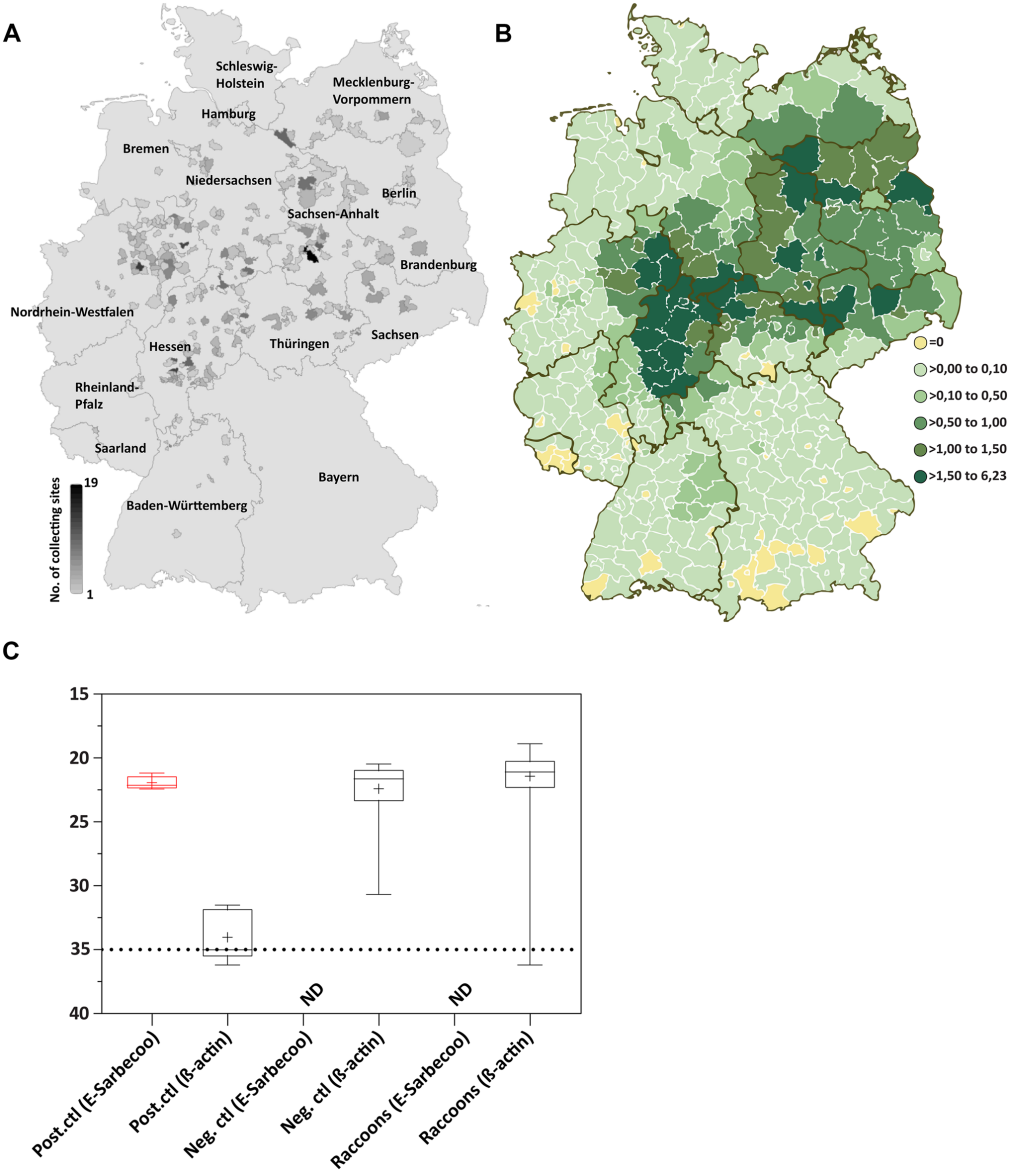
RT-qPCR-based surveillance on wild raccoon populations to monitor natural infections by SARS-CoV-2 in Germany. **A** Schematic depiction of collection sites for the analyzed raccoon samples across Germany on county level. The more samples collected from certain site, the more opacity of the color (maximum capture places were 19 and were from Sachsen-Anhalt). **B** Representing the hunting ranges of raccoons in pieces/100 ha huntable area (reproduced with kind permission of WILD-Monitoring/Deutscher Jagdverband), samples were taken in areas with high hunting ranges. **C** Results of RT-qPCR represented by Ct values for E-Sarbeco and β-actin as a negative control for RNA. Positive (cell culture supernatants) and negative (clinical samples) controls were used across all the examined samples

Here we collected 820 lung tissue samples from hunted raccoons mainly coming from four different German federal states representing regions with high hunting ranges (Saxony-Anhalt, Thuringia, Hesse, North Rhine-Westphalia; Fig. 1A–B) and examined them by reverse transcriptase real-time polymerase chain reaction (RT-qPCR). We selected these animal species because they are common in urban areas and therefore come into contact with the human population either through consumption of garbage or directly in wildlife rehabilitation centers or even zoos. The tissue samples were homogenized (TissueLyser II, Qiagen, Germany) and viral RNA was extracted (MagMax, Invitrogen; Thermo Fisher Automated Nucleic Acid Extraction Workstation). Extracted RNA was further examined by RT-qPCR using an *envelope* (E) gene and β-actin targets and CFX real-time PCR systems (Bio-Rad, Germany) as previously described (Corman et al. 2020). Next, we analyzed why these species are rather resistant to SARS-CoV-2 infections, both in experimental and natural settings (Francisco et al. 2021). We used bioinformatics and structural approaches (Geneious Prime® 2021.0.1.) to compare essential ACE-2 residues in raccoons and humans, and in species known to be fairly resistant (poultry and pigs).

## Results

**Fig. 2:**
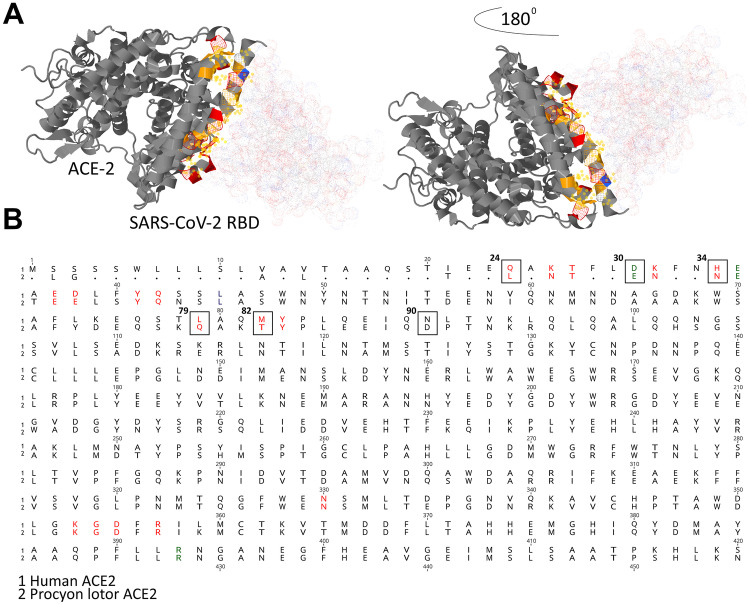
Interactions of SARS-CoV-2 RBD, human and raccoons ACE2. **A** The amino acids that are essential for this interaction are shown in yellow. The figure was created by Geneious Prime® 2021.0.1. **B** Multiple-alignment of human and raccoons ACE-2, highlighting the difference in important residues for binding to SARS-CoV-2 receptor binding domain (RBD). Residues in red represents amino acids essential for binding of SARS-CoV-1 and SARS-CoV-2, while in green represents those essential for the contact of SARS-CoV-2 only. Residues in boxes are those substituted in *Procyon lotor* species and other SARS-CoV-2-resistant animal species

No SARS-CoV-2 specific viral RNA was detected in any of the examined animals (0/820) (Fig. 1C). Based on our results, wild raccoons are unlikely to be a reservoir or amplifying host for SARS-CoV-2. SARS-CoV-2 possess a trimeric structure called spike protein that determines cell and host tropism. Establishment of the infection requires a cleavage of S-protein by ubiquitous host proteases to S1, which contains the receptor binding domain (RBD), and S2, a fusion protein. Binding of the RBD to the host cellular receptor, angiotensin converting enzyme 2 (ACE2) is a prerequisite for viral infection. Zhai et al. proposed certain residues in ACE-2 receptors that are essential for binding to the viral RBD and determining virus host range and postulated that animals with more amino acid changes at the ACE2-S interface are at lower risk for SARS-CoV-2 infection (Zhai et al. 2020). Here we detected only 60% identity (Fig. 2B) between residual ACE-2 in humans and raccoons, which may explain the lack of susceptibility to SARS-CoV-2 in these species. Among these deviant amino acids are Gln24 that forms a hydrogen bond to S N487, His34 in the center of the binding site, Asp30 that forms the central salt bridge, Leu79 and Met82 that interact with Phe486 in the peripheral binding sites, and the N-glycosylation site Asn90. These amino acids were substituted in ACE2 of pigs (Q24L, D30E, H34L, L79I, M82T, N90T) and chickens (Q24E, D30A, H34V, L79N, M82R). Pigs and chickens are known to be resistant to SARS-CoV-2 (Vergara‐Alert et al. 2021; Suarez et al. 2020; Michelitsch et al. 2021). Interestingly, we have found that these five amino acids were also substituted in ACE-2 of raccoon species (Fig. 2B). For instance, the Gln24 was substituted to Leu and therefore hydrogen bonds cannot be formed. Also, the Asp30 residue was swapped to Glu that is negatively charged and therefore the salt bridge to S Lys417 remains intact. Moreover, the His34 that interact with Tyr453, Leu455, and Gln493 in the interaction surface is replaced by Asn. Further, the Leu79 that interacts with S Phe486 was replaced to Gln and also the Met82 to Thr. More importantly, the N-glycosylation site Asn90 was deleted due to replacement by Asp.

## Conclusion

Together, our data are consistent with previous reports about the susceptibility of raccoons to SARS-CoV-2 and highlight that raccoons do not function as competent reservoirs for SARS-CoV-2 (Francisco et al. 2021). Since raccoons often living in close neighborhood to humans not only in rural areas but also in regions with high urbanization, they represent a source of zoonotic diseases that should not be underestimated. That reservoirs for SARS-CoV-2 can form in wildlife populations has been demonstrated by transmissibility and spread studies with mink and white-tailed deer (Hale et al. 2021). Wild individuals of these species susceptible to SARS-CoV-2 have been shown to transmit the virus within wild populations and can also transmit it to kept conspecifics or even humans when contact occurs (Munnink et al. 2021; Pickering et al. 2022). Surveillance projects to monitor such populations are necessary to quickly detect and control unrestraint spread of the virus. Such a project was launched in the European framework for monitoring mustelids (European Food Safety Authority et al. 2021). Other European wildlife species must also be considered to represent potential host for the virus (Delahay et al. 2021), e.g., bats, carnivores, and cervids. Especially those susceptible species with close relationships to other species like raccoon dogs and raccoons deserve a special attention. Further studies including other carnivorous animals, rodents, and others regarding distribution of the virus and receptor studies must be done in the future to show as comprehensive a picture as possible of the spread of SARS-CoV-2 in wildlife species. The results of such studies must be brought together for a superior valuation and coordination of necessary responses. The present study shows that raccoons, including those living in close proximity to humans, are currently not expected to pose an increased risk for humans and other wildlife species. However, in view of the constant emergence of new variants of the virus, it seems necessary to include raccoons in surveillance studies. In this context, attention should be paid to variants and selective alterations in the genome of the viruses that facilitate binding of the virus to the cellular receptors of the different species.
